# Efficient Soil Temperature Profile Estimation for Thermoelectric Powered Sensors

**DOI:** 10.3390/s25134232

**Published:** 2025-07-07

**Authors:** Jiri Konecny, Jaromir Konecny, Kamil Bancik, Miroslav Mikus, Jan Choutka, Jiri Koziorek, Ibrahim A. Hameed, Algimantas Valinevicius, Darius Andriukaitis, Michal Prauzek

**Affiliations:** 1 Department of Cybernetics and Biomedical Engineering, VSB—Technical University of Ostrava, 17. Listopadu 2172/15, 708 00 Ostrava-Poruba, Czech Republic; jiri.konecny@vsb.cz (J.K.); kamil.bancik@vsb.cz (K.B.); miroslav.mikus@vsb.cz (M.M.); jan.choutka@vsb.cz (J.C.); jiri.koziorek@vsb.cz (J.K.); michal.prauzek@vsb.cz (M.P.); 2 Department of Mechanical Engineering and Technology Management, Norwegian University of Life Sciences (NMBU), Drøbakveien 31, 1433 Ås, Norway; ibrahim.abdelhameed@nmbu.no; 3 Department of Electronics Engineering, Kaunas University of Technology, K. Donelaicio g. 73, 44249 Kaunas, Lithuania; algimantas.valinevicius@ktu.lt (A.V.); darius.andriukaitis@ktu.lt (D.A.)

**Keywords:** energy harvesting, Internet-of-Things sensors, long short-term memory, polynomial regression, support vector regression, temperature modelling

## Abstract

Internet of Things (IoT) sensors designed for environmental and agricultural purposes can offer significant contributions to creating a sustainable and green environment. However, powering these sensors remains a challenge, and exploiting the temperature difference between air and soil appears to be a promising solution. For energy-harvesting technologies, accurate soil temperature profile data are needed. This study uses meteorological and soil temperature profile data collected in the Czech Republic to train machine learning models based on Polynomial Regression (PR), Support Vector Regression (SVR), and Long Short-Term Memory (LSTM) to predict the soil temperature profile. The results of the study indicate an error of 0.79 °C, which is approximately 10.9% lower than the temperature error reported in state-of-the-art studies. Beyond achieving a lower temperature prediction error, the proposed solution simplifies the input parameters of the model to only ambient temperature and solar irradiance. This improvement significantly reduces the computational costs associated with the regression model, offering a more efficient approach to predicting soil temperature for the purpose of optimizing energy harvesting in IoT sensors.

## 1. Introduction

With the advent of advanced IoT sensors, the field of environmental and agricultural monitoring is experiencing an explosion of growth. The application domains work with numerous challenges, including forest fires, drought, and climate change [1]. To address these challenges effectively, IoT technology must overcome several obstacles, including consistent power delivery, reliability, and resilience to adverse environmental conditions [1]. Resolving power delivery issues for autonomous IoT sensors, especially sensors deployed in locations where connection to the power grid is unfeasible and regular maintenance, such as battery replacement, is prohibitively expensive or even impossible due to the remoteness of the location or the complexity of installation, is highly challenging [2].

Often positioned on the ground, environmental and agricultural sensors can benefit from energy-harvesting thermoelectric generators (TEG) that exploit the temperature differential between air and soil [3]. A critical challenge is the seasonal variability in harvested energy, necessitating the use of intelligent energy management strategies such as machine learning for developing sophisticated models that control measurement intervals and data transmission [4]. To enhance the efficiency and designs of such models, access to soil temperature data is imperative. Consequently, estimating the soil temperature profile is essential in designing cost-effective and reliable IoT sensors.

**Figure 1:**
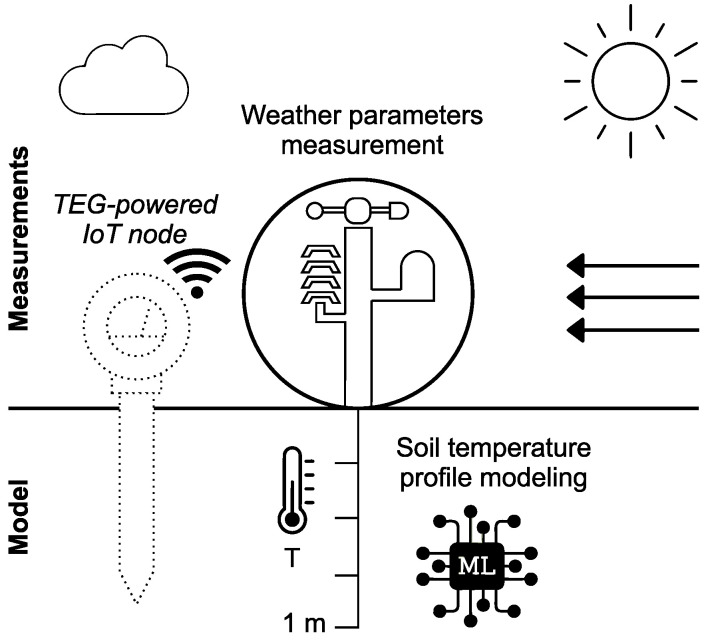
Diagram illustrating weather parameters as inputs to a model designed for estimating the soil temperature profile, thereby simulating the behaviour of a thermoelectric generator-powered IoT sensor.

Figure 1 presents a diagram of the input parameters acquired by a weather monitoring station. These parameters are used in estimating the soil temperature profile with the model proposed in the present study. Figure 1 also demonstrates the application of the derived profile in simulating a thermoelectric generator-powered IoT node.

Table 1 provides a comprehensive overview of related studies that both underpin and enhance the findings of the research presented here. Primarily, the studies are concerned with the prediction of soil temperature, a critical factor in environmental monitoring and agricultural practices. The table also lists studies that explore the application of soil temperature differentials for the generation of green energy and highlight the innovative use and potential of TEGs in sustainable power solutions for remote sensor networks. A study which examines the impact of soil temperature variations on biodiversity especially enriches the discussion by offering insight into the broader ecological implications of temperature changes. This multifaceted approach not only underscores the significance of accurate soil temperature modelling but also illustrates the diverse applications and environmental considerations linked to this research domain. By integrating these varied perspectives, the table serves as a foundational element for guiding the development of sophisticated models and strategies that provide solutions to the challenges posed by climate change and the need for sustainable agricultural and environmental monitoring platforms.

**Figure 2:**
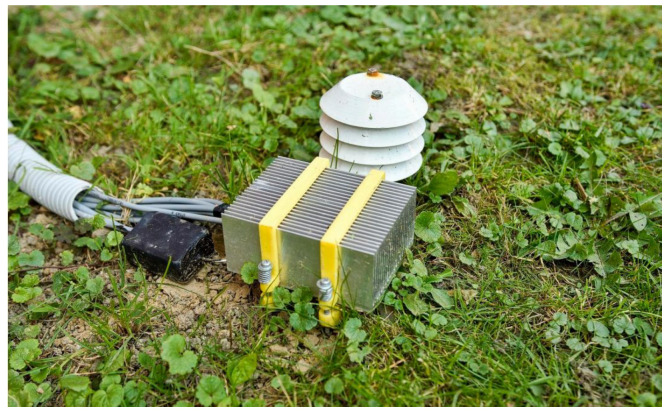
The energy-harvesting device deployed at the experiment site.

Although the primary aim of this study is to estimate soil temperature profiles for energy simulation purposes, thermoelectric generators (TEGs) have already been successfully employed in a variety of practical IoT sensing systems. For instance, ref. [10] presented a self-powered wireless monitoring system for photovoltaic modules, where residual heat was harvested to power a Bluetooth-enabled temperature sensor. Similarly, a greenhouse environment was explored in [11], where nineteen TEG modules converted the temperature gradient between circulating water and greenhouse air into usable energy for powering IoT sensors with supercapacitor buffering. Another approach is described in [2], which demonstrated how phase change materials can stabilise thermal gradients and maintain continuous power generation in fluctuating ambient conditions. Broader applications of TEG-powered sensors—such as for wearable devices, structural monitoring, and aviation—are reviewed in [12], highlighting the expanding relevance of TEGs in the Internet of Things domain. Figure 2 presents a demonstration of an agricultural IoT sensor powered by a thermoelectric generator (TEG). The device is positioned above ground level, allowing the heat sink to be exposed to ambient air for an optimal thermal gradient.

Based on the state of the art, the present study contributes with the design of an estimation model that satisfies the following criteria:

1. The model enables the estimation of soil temperature profiles based on fundamental weather parameters;
2. The model demonstrates the capability to estimate detailed dynamic temporal behavior within the soil temperature profile.

The rest of the text is organised as follows: Section 2 introduces the appropriate machine learning methods and models for soil temperature profile estimation and also defines the datasets, evaluation criteria, and experimental methodology applied in the study; Section 3 presents the experimental results, including exploratory data analysis, feature cross-validation, model comparison, and time-domain analysis; Section 4 compares the proposed model with state-of-the-art studies; finally, Section 5 concludes the article.

## 2. Methods and Models

This section outlines the methods and models employed for predicting soil temperature. It also details the dataset, experimental procedure, and evaluation criteria applied in the present study.

### 2.1. Methods

Various machine learning methods can be used to predict temperature in the soil. These methods input meteorological data into an LSTM model, which is then compared with other machine learning approaches.

Table 2 presents an overview of the machine learning models reviewed in this article. Polynomial Regression (PR) is the most commonly employed prediction model and serves as a reference model. In the present study, PR uses a Linear Regression model enhanced with polynomial preprocessing to enable the processing of non-linear data [13]. SVR, from the Support Vector Machine family, is another widely employed algorithm that is expected to yield satisfactory results. SVR is noted for its favorable balance between computational costs and performance and its capability to handle multidimensional problems [14]. The LSTM neural network is the most versatile and generative of the approaches mentioned, offering extensive optimisation possibilities in terms of structure, penalties, and cost functions. Generally, LSTM is known to achieve lower error rates [15] and is effective in time-series analysis problems [16].

The configuration of PR and SVR models also depends on the number of input parameters. For PR, the degree of polynomial features must be established. In the present study, the degree range is set between one and ten. In the case of SVR, the regularisation parameter (*C*) and the insensitivity loss parameter (ϵ) are selected. The regularisation parameter is set within a range of 1 to 100, and the insensitivity loss parameter is set within a range of 0.01 to 0.1.

**Figure 3:**
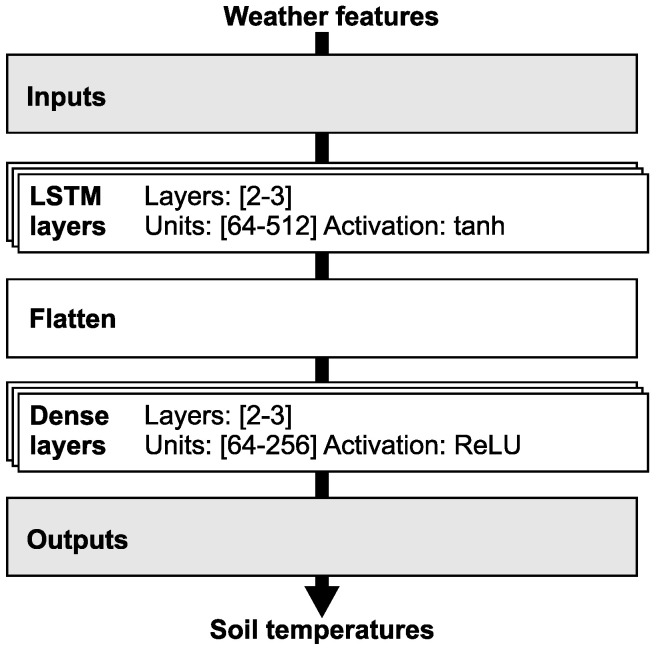
LSTM model structure: Main structure, layers, activation function definitions, inputs and outputs.

The LSTM model is a general framework that, for the purpose of soil temperature prediction, requires specific configurations in its structure, loss function, and optimizer. Figure 3 illustrates the LSTM model structure adapted for use in the presented experiments. The figure outlines the main LSTM components, including its structure, layer types, activation function definitions, inputs, and outputs. LSTM layers process input data, identify patterns, and manage time series data. Dense layers translate the output from LSTM layers into output vectors. Inputs are derived from the model’s feature sets, while outputs directly correspond to the soil temperature profile at various depths. The architecture of the LSTM neural network is dependent on the number of features in its feature set; naturally, more features necessitate a more complex neural network. Based on the number of features, the number of layers and neurons is adjusted. To prevent overfitting, the model employs L1 and L2 penalties and dropout techniques.

The LSTM model uses up to seven feature sets. A soil temperature profile at four depths is predicted for each feature set.

Table 3 summarises the parameters and respective values or ranges for the LSTM model. To optimise the performance for each feature set, a bespoke model was carefully constructed to minimise loss and error rates. This approach produced seven individual LSTM model configurations, each tailored to the specifics of its corresponding feature set. The ranges listed for certain parameters in Table 3 describe the variability and adaptability required to fine-tune the models for optimal performance.

Notably, all model configurations use the same optimiser, loss function, observed metrics, and output layer configurations. This consistency ensured a standardised approach to training and evaluating the model and permitted a coherent comparison of performance metrics across the different LSTM models. In adopting this rigorous and tailored methodology, the present study not only improves on the accuracy and reliability of soil temperature predictions, it also provides valuable insight into the effective use of LSTM networks in complex data-driven forecasting tasks.

Although many data-driven methods exist for time-series regression and environmental modelling, including Random Forest Regression (RFR), Gradient Boosting Machines (GBM), Gaussian Process Regression (GPR), or more recent Transformer-based architectures, the selection of PR, SVR, and LSTM in this study was based on a balance of model interpretability, computational cost, and prior success in similar soil temperature prediction tasks. Polynomial Regression offers a simple and interpretable baseline; SVR is known for its robustness in small-to-medium datasets with non-linear structure; and LSTM has become a standard for capturing temporal dependencies in multivariate time series. This combination allows for comparing classic regression, kernel-based learning, and deep learning approaches within a unified framework, while maintaining accessibility for deployment and further development.

### 2.2. Data

The dataset for the experiments consists of meteorological variables recorded at 10-minute intervals. This dataset was professionally recorded at the Ostrava-Poruba station in the Czech Republic and acquired from the Czech Hydrometeorological Institute (CHMI); it includes weather data such as temperature, solar irradiance, precipitation, air pressure, and soil temperature. The data cover a period of four years (2016–2019) and contain a soil temperature profile used for evaluation of the experimental results. The dataset can be accessed by contacting the CHMI [21].

Table 4 lists the variables contained in the input dataset. The variables were recorded at 10 min intervals and specifically describe wind speed (*F*), atmospheric pressure (*p*), solar irradiance (*S*), precipitation (*R*), ambient temperature (*T*), and soil temperature ($T_{xxx}$) at depths of 5 cm, 10 cm, 20 cm, 50 cm, and 100 cm. Because temperature in soil changes slowly and inertially and does not experience the same rapid changes as ambient heat, wind or solar irradiance, the dataset was resampled to a one-hour interval using an averaging window function.

### 2.3. Evaluation Criteria

The experiments were evaluated according to several criteria essential to determining both the performance accuracy and error rate in the LSTM models. These criteria were also crucial to evaluating the effectiveness of input parameter combinations and provided insight into the effects of the variables on prediction accuracy.

Table 5 lists the evaluation criteria for the experiments, including abbreviations and units. MAE, RMSE, and $R^{2}$ indicate the statistical properties of the presented results. Adapted from the MAE and RMSE, Error Ratio represents the model’s weighted error ratio. The Total Score is calculated from the Error Ratio and $R^{2}$ and represents a measure of the model’s quality.

For assessing the predictive model’s performance, MAE and RMSE are key criteria for quantifying the average deviation of predicted outcomes from their actual values, expressed in degrees Celsius. The coefficient of determination, $R^{2}$, quantifies how well the model’s predictions match the variability of the observed data and ranges from 0 to 1, where 0 indicates no explanatory power and 1 indicates complete agreement between the model’s predictions and measured results.

Building on these traditional metrics, the present study introduces Total Score, a composite metric derived from the Error Ratio and $R^{2}$ coefficient. This score synthesises the insights gained from error metrics and $R^{2}$ coefficient into a single, comprehensive evaluation metric ranging from 0 to 100.

To calculate the Total Score of a model, it is necessary to first compute its Error Ratio, which is obtained from the equation:

(1) $$\mathrm{ER}=\frac{w_{\mathrm{MAE}}\cdot \left(1-{\mathrm{MAE}}_{\mathrm{rel}}\right)+w_{\mathrm{RMSE}}\cdot \left(1-{\mathrm{RMSE}}_{\mathrm{rel}}\right)}{\sum (w)}\cdot 100,$$

where ER—Error Ratio is the relative error of the model in the range 0 to 100, $w_{\mathrm{MAE}}$ and $w_{\mathrm{RMSE}}$ are the weight coefficients in the range 0 to 1 (the sum of of the weights = 1), and ${\mathrm{MAE}}_{\mathrm{rel}}$ and ${\mathrm{RMSE}}_{\mathrm{rel}}$ are error metrics transformed to their relative forms according to the equations:

(2) $${\mathrm{MAE}}_{\mathrm{rel}}=\frac{\mathrm{MAE}}{\max\left(T_{xxx}\right)-\min\left(T_{xxx}\right)},$$

(3) $${\mathrm{RMSE}}_{\mathrm{rel}}=\frac{\mathrm{RMSE}}{\max\left(T_{xxx}\right)-\min\left(T_{xxx}\right)},$$

where $\max(T_{xxx})$ and $\min(T_{xxx})$ are soil temperatures ($T_{xxx}$). This normalisation step ensures that the Error Ratio reflects the weighted contributions of both MAE and RMSE relative to the total weight. The experiment used weights of 0.2 for MAE and 0.8 for RMSE. The normalised Error Ratio is then scaled by a factor of 100 for conversion into a percentage. Finally, a Total Score is calculated:

(4) $$\mathrm{Total}\;\mathrm{Score}=\left(100-\mathrm{ER}\right)\cdot R^{2}$$

The Total Score metric, which is critical to evaluating the model, ingeniously combines assessment of the error ratio and the data variance. It provides a comprehensive measure of the model’s ability to explain the data variance and predictive accuracy by combining the error ratio to reflect the model accuracy and the $R^{2}$ value to describe the variance. A high Total Score indicates the model’s efficiency in both aspects, indicating superior performance.

Although the current evaluation is based on aggregated metrics such as MAE, RMSE, and a composite Total Score, alternative multi-criteria decision-making (MCDM) approaches, such as the Technique for Order of Preference by Similarity to Ideal Solution (TOPSIS), could also be applied. These methods may offer complementary insights when comparing models across multiple evaluation dimensions, especially in scenarios where trade-offs between different performance metrics are important. Exploring such approaches could be a subject of future research, particularly for model selection under uncertainty or deployment constraints.

### 2.4. Experimental Methodology

This section provides a detailed overview of the experimental process and outlines the comprehensive methodology used to achieve the study’s aims. The primary aim of the experiment was to identify the optimal feature set and machine learning model for predicting soil temperature using the supplied dataset. This involved not only careful selection and evaluation of various prediction models but also careful identification of the features relevant and instrumental to soil temperature prediction accuracy.

**Figure 4:**
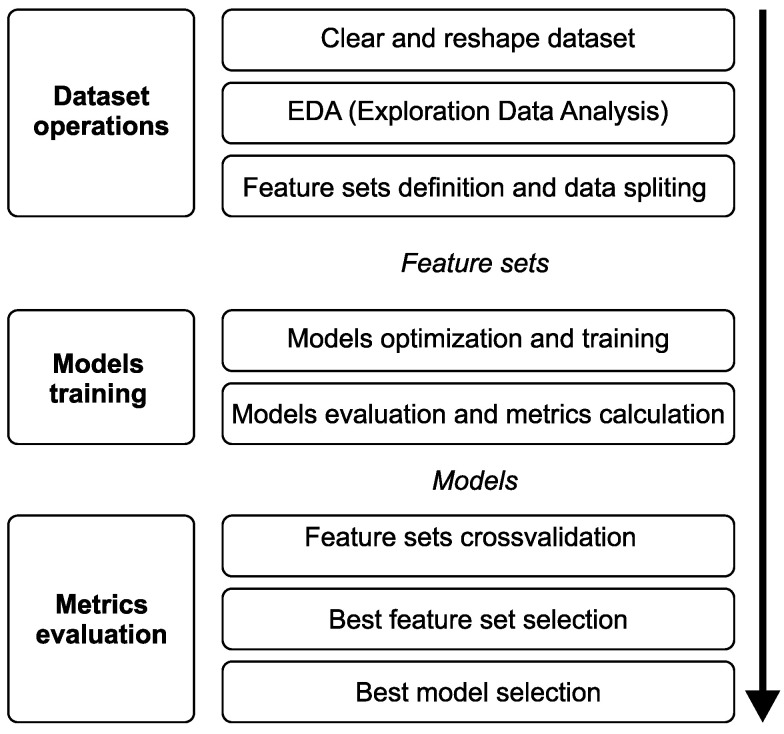
Diagram of the experimental workflow and specific steps.

Figure 4 details the workflow and specific steps of the experiment. The procedure began with an Exploratory Data Analysis (EDA), a phase essential to acquiring a deep understanding of the characteristics of the dataset and the intricate patterns it contains. This initial analysis was critical to revealing the data structure, discovering potential correlations, and identifying any anomalies or outliers with a potential impact on the study’s results. After the EDA, the feature sets were identified and prepared. These sets were selected carefully according to their relevance and potential impact on the predictive capabilities of the models and served as the building blocks for creating other models with improved accuracy and predictive power.

The next phase of the experiment involved producing a detailed design and fine tuning and testing three predictive models (PR, SVR, and LSTM) on each of the selected feature sets. This phase identified the most effective configurations and performed a thorough search for the optimal hyperparameters of each model. Adjustments were made according to the specific feature set by creating a parameter grid of different suitable hyperparameter combinations for each model, and for each of these combinations, the model was trained and tested on a small fraction of the real dataset. The results were then processed, and the combination which produced the lowest error rate was selected for additional processing. Each model was then fine-tuned, followed by testing and calculation of the models’ metrics. This systematic approach enabled a comprehensive evaluation of each model’s predictive accuracy, and crucially, its ability to generalise to unseen data.

Finally, the experiment moved into a cross-validation phase where each feature set was compared to determine the most effective combination and best respective predictive model. This phase examined the suitability of each feature set from multiple analytical perspectives, including a comprehensive comparison of the overall average total score, identification of the highest total score, assessment of the effectiveness of the feature set at different soil depths, and a detailed analysis of both the average and highest total scores, specifically at the 50 and 100 cm depths. This multi-faceted assessment provided an overall understanding of the predictive power of each feature set and its impact on model performance in different scenarios. Once the most appropriate feature set was identified, the best overall model with the highest total and average achieved jump was selected.

## 3. Results

This section presents detailed results of the experiment and discusses the findings of each of its steps, beginning with the EDA through to the final cross-validation of input feature sets and evaluation of individual models’ error rates and efficiencies. The effectiveness of each feature set and model is analysed, using a range of metrics to assess performance. Time-based soil temperature prediction graphs are also presented as visual representations of the accuracy and reliability of the selected model.

### 3.1. Exploratory Data Analysis

The EDA analysis attempted to determine the relationships between the meteorological parameters and soil temperatures measured in the experiment. The analysis also removed outliers from the tested dataset.

**Figure 5:**
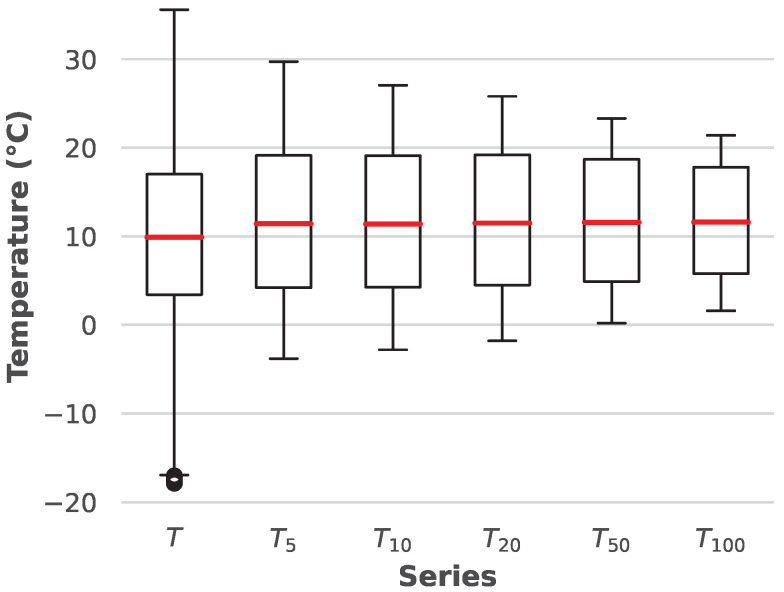
Box plot of environmental temperatures and soil temperatures.

Naturally, it is expected that the soil temperature is closely related to the ambient temperature. Figure 5 presents the statistical data of the measured ambient temperatures and soil temperatures at various depths. Notably, the median ambient temperatures are nearly identical to the median soil temperatures at all soil depths. The distribution of the box plots (25th and 75th percentiles) is also similar, and as depth increases, the range of values narrows, indicating that temperature becomes more stable at greater depths throughout the year.

**Figure 6:**
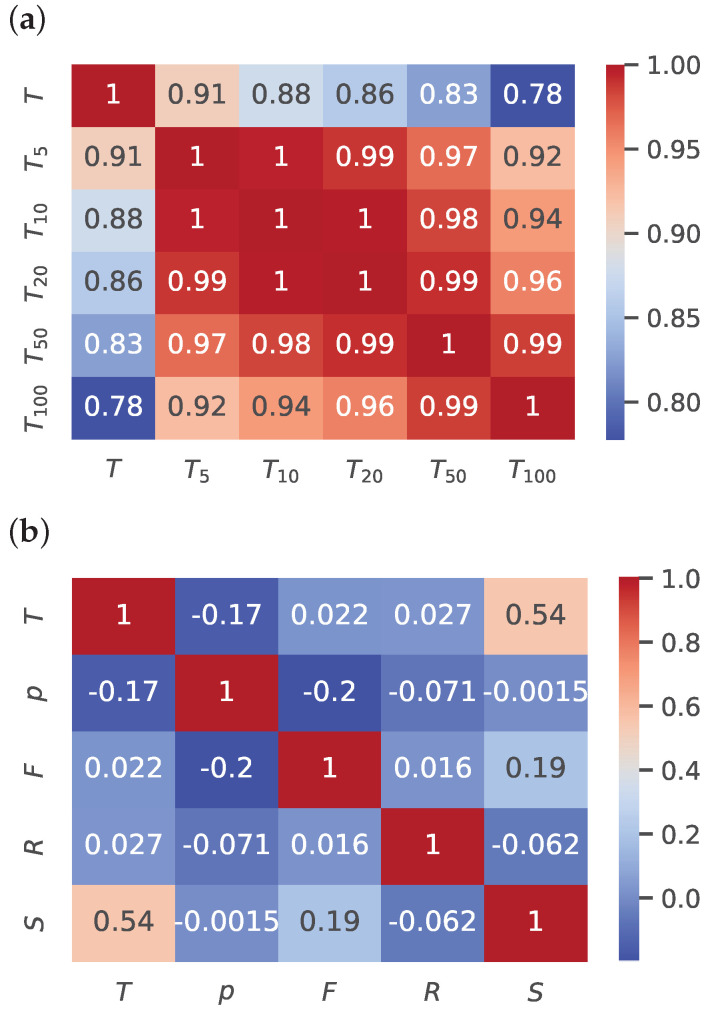
(**a**) Air and soil temperature correlation heat map. (**b**) Weather conditions and air temperature correlation heat map.

Figure 6a illustrates the correlation between ambient temperature and soil temperature as a heat map. The correlation coefficients are very high, indicating a strong relationship between ambient and soil temperatures.

Figure 6b depicts the correlation between various weather parameters as a heat map. The heat map identifies the meteorological factors which have a significant effect on ambient temperature. In this case, ambient temperature is strongly affected by the intensity of solar irradiance. Additionally, ambient temperature has a weak negative correlation with atmospheric pressure and an almost negligible correlation with precipitation and wind. From these observations, it can be concluded that ambient temperature in conjunction with solar irradiance, and to a lesser extent, atmospheric pressure, have a significant effect on soil temperature. This conclusion is logical since solar irradiance is the source of most energy that reaches the Earth’s surface to affect soil temperature. Other factors have minor roles, with primarily direct or indirect effects on the intensity of solar irradiance (e.g., clouds, rain) and ambient temperature (e.g., heat absorption).

Based on these observations, eight feature set combinations were proposed. Table 6 lists the feature set combinations selected for evaluation. All combinations, apart from the first, which uses only ambient temperature, include ambient temperature and solar irradiance due to the very high correlation of these parameters with soil temperature.

### 3.2. Feature Cross-Validation and Best Combination

This section presents a performance analysis of the selected combinations of feature sets. The analysis identified the most effective feature set combinations suitable for predicting soil temperatures. All feature set combinations were tested using the PR, SVR, and LSTM models for a soil depth of 100 cm.

**Figure 7:**
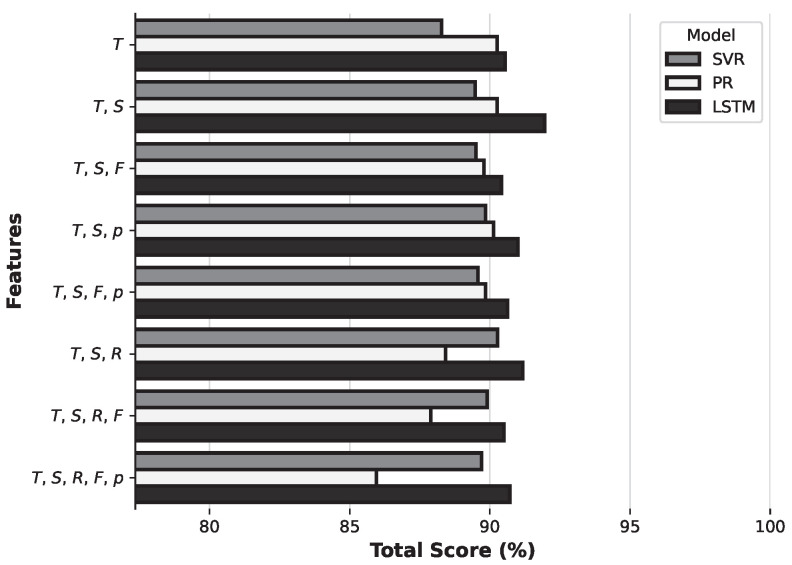
Comparison of feature sets according to the total score obtained for 100 cm soil depth.

Figure 7 compares the various feature set combinations tested with the PR, SVR, and LSTM models. For each combination, the total score is relatively high, with the following observations. For combinations of two and three features, PR achieved a higher score than SVR. For combinations with more than three parameters, SVR yielded higher performance. LSTM consistently achieved the highest score in all feature set combinations. Notably, the combination of ambient temperature and solar irradiance achieved the highest total score.

**Figure 8:**
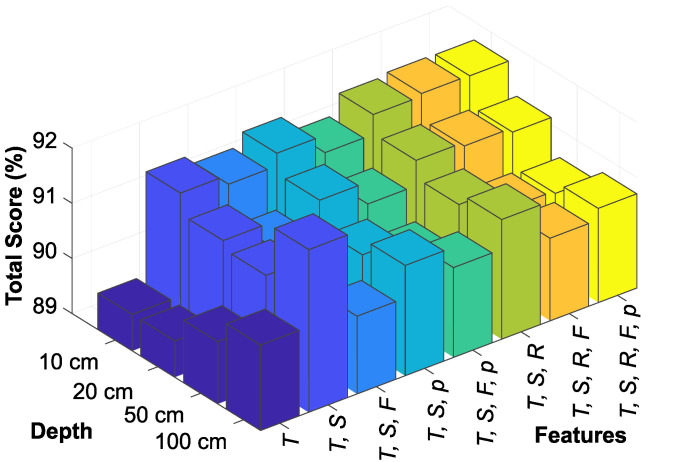
Comparison of feature sets according to soil depth for the LSTM model.

Figure 8 graphs the effectiveness of various feature sets at different soil depths, using the LSTM model to evaluate the total score for each set. The combination of ambient temperature and solar irradiance was the most effective predictor at all depths, its dominance especially evident at 100 cm soil depth. However, the prediction score at 50 cm soil depth indicates a decreasing trend and suggests a transition layer that potentially challenges the model’s accuracy. In addition, the inclusion of wind as a feature negatively affected the model’s predictive ability, highlighting the criticality of selecting suitable features in enhancing the accuracy of soil temperature predictions.

The analysis revealed a notable decrease in prediction accuracy at a soil depth of 50 cm for all tested models. This depth approximately delineates the boundary between frozen and non-frozen soil layers at the studied location, serving as a transitional zone. Above this depth, soil layers tend to be more thermally unstable and directly affected by weather changes, exhibiting lower thermal inertia. Conversely, below this depth, the soil layers become more thermally stable, benefiting from higher thermal inertia due to improved insulation from external weather conditions. This transition zone, marked by significant thermal variability, poses a challenge for predictive models by impacting their ability to accurately forecast soil temperatures at this intermediate depth.

### 3.3. Comparison of Models

This section presents a comparative analysis of the models, using the highest-performing feature set (ambient temperature and solar irradiance). Individual models were compared mainly on total score metrics, but the traditional metrics MAE, RMSE, and $R^{2}$ were also examined.

**Figure 9:**
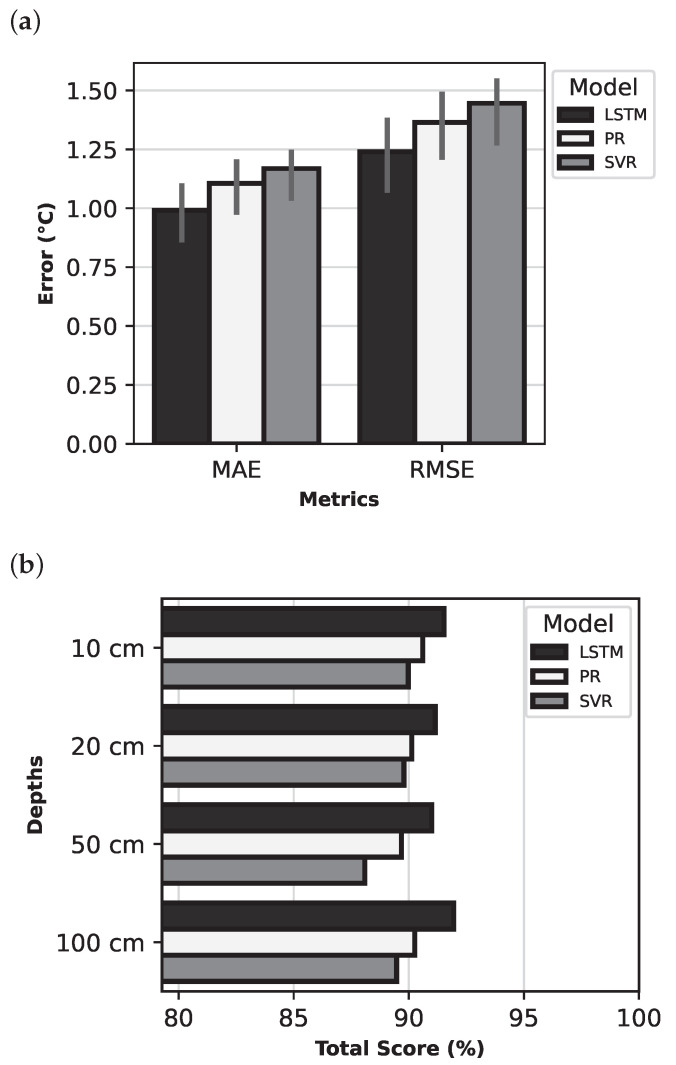
Errors and best feature set scores for the models: (**a**) Comparison of MAE and RMSE; (**b**) Comparison of Total Score at various depths.

Figure 9 provides an overall comparison of the models in terms of (a) the standard metrics MAE and RMSE and (b) the total scores of the models at different soil depths. For both MAE and RMSE, the LSTM model achieved the lowest values, indicating superior performance. The graphs also depict the variability in the MAE and RMSE criteria. The line at the top of the bars represents the minimum and maximum error values, varying with depth. The LSTM model attained the highest total score, which aligns with previous observations.

Table 7 summarises the metric-specific values for the models evaluated at various soil depths (10–100 cm). All models, across all depths, achieved similar results for $R^{2}$, indicating that the statistical properties of the predicted temperatures closely resemble those of real-life data. The results also demonstrate that all of the models are suitable for predicting soil temperature profiles, with relative errors in the range of approximately 5–6%. The LSTM model achieved the best performance, especially with MAE in the range 0.79–1.10 °C and RMSE in the range 0.98–1.38 °C. The accuracy of the LSTM model increased with depth.

### 3.4. Time-Domain Analysis

This section compares the LSTM model’s ability to predict soil temperatures at different depths with real data for the best feature set combination of ambient temperature and solar irradiance.

**Figure 10:**
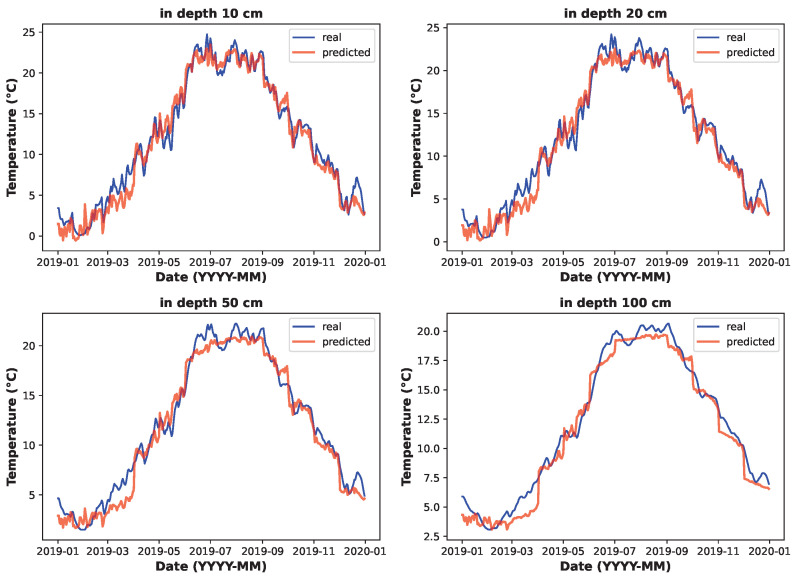
Predicted soil temperature versus real temperature at various depths (LSTM model).

Figure 10 compares the LSTM model’s predicted soil temperature data with a historical sample of soil temperature data (2019). The graphs indicate that the predicted data and real data share a similar character. Based on the observations and error values discussed above, it can be stated that the prediction model performed adequately and is suitable for its purpose.

**Figure 11:**
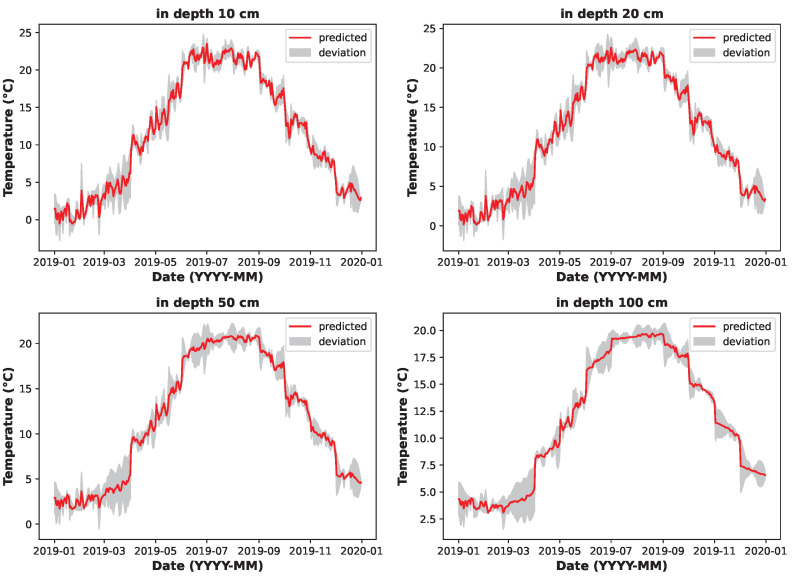
Predicted soil temperature deviation at various depths (LSTM model).

Figure 11 graphs the error deviations of predicted values over the course of one year (2019). The theoretical maximum deviation from the real data (grey) occurs in the spring season (March to April) since weather conditions change rapidly during this period and thus complicate the accurate prediction of soil temperatures. The smallest deviations, however, occur in the summer season, when the weather is generally more stable.

## 4. Discussion

This section reviews the computational costs of the proposed models and discusses the benefits and limitations of the proposed solution in relation to state-of-the-art methods.

Generally, the PR model demonstrated the lowest computational costs, while the SVR and LSMT models were up to 20 times higher. Most of the computational power was required during the training phase and for determining optimal hyperparameters. During the prediction phase, the PR model also demonstrated lower computational costs than the SVR and LSMT models. However, compared to the learning phase, the computational power required for calculating predictions was negligible. The proposed models were executed on the Python 3 Back-end Google Compute Engine in the Google Colab online environment. For training, the PR model required approximately one minute, while the LSMT and SVR models required around 20 min.

Table 8 compares the results of the present study with the SOTA method published in [5]. The proposed solution and the reference study apply similar methods (PR, SVR, LSTM) and evaluation criteria, thus enabling a straightforward comparison. Despite the proposed approach using only two feature sets (i.e., reference temperature and solar irradiance), the total error is lower than the error in the reference study, which uses 12 feature sets. Compared to the reference solution, the MAE and RMSE errors are lower (i.e., better), and $R^{2}$ is higher (i.e., also better). This suggests that this study optimises models more efficiently. In general, the proposed solution improved on the results in the reference study by 10.9%.

The proposed model’s main advantage is the use of only two parameters (ambient temperature and solar irradiance) for prediction and thus eliminating the need for data for more advanced parameters. Data for these two parameters are common and readily available from weather stations. Soil profile estimation is a useful tool for designing IoT sensors that effectively harvest power from the temperature differentials existing between ambient air and soil. Moreover, the proposed model predicts the trend in temperature over time instead of the daily sum, which is useful for IoT sensor simulations [4]. Importantly, the model is not intended for deployment on the sensor node itself. Instead, it serves as a decision-support tool for the offline design and simulation of thermoelectric energy harvesting behaviour in locations without direct soil temperature measurements. Although the soil temperature profile is essential for estimating TEG energy yield, it does not actively influence the power management strategy of the sensor node. Instead, the predicted profile is used during the design and planning phase to simulate energy availability under various environmental conditions.

The present study’s main drawback is it uses results from an experiment performed at a single location only. It cannot be clearly stated how the model would behave under different climatic conditions at another location. The model was trained on a dataset that spans three years of measurements and then tested and evaluated on a one-year fragment, which does not accurately represent the model’s functioning over a long-term horizon.

## 5. Conclusions

The present study developed several models capable of predicting the soil temperature profile, which is a crucial element for advanced algorithms that control IoT sensors powered by thermal energy harvesters. Understanding the soil temperature trend improves the results of sensor deployment simulations and assists in optimising energy management strategies for use in IoT sensor designs.

A prediction model was developed to enable calculation of the soil temperature profile up to a depth of one metre. The models, based on PR, SVR, and LSTM neural networks, applied common meteorological parameters. Overall, the proposed solution improved on the results of a reference study by approximately 10.9%. The findings also indicate that ambient temperature and solar irradiance data are sufficient for high-accuracy prediction of the soil temperature profile.

Future work will involve performing the experiment at other locations to validate the findings. The models can be deployed to work with TEG devices and provide the basis for a comprehensive tool for predicting soil temperature and creating soil temperature profiles for any geolocation based on weather forecasting or historical data.

## Figures and Tables

**Table 1:** Summary of related studies on soil temperature prediction and its applications.

Article	Author, Year	Relevant Content
Modelling hourly soil temperature [5]	Cong Li, 2020	Combination of hourly and daily soil temperature prediction up to 100 cm depth High-resolution model for agriculture or geothermal applications Application of a large dataset from diverse climates Comparison of traditional vs. deep learning model
Spatio-temporal modelling for soil temperature prediction [6]	Xiaoning Li, 2023	High accuracy and interpretability Use of Convolutional and Long Short-Term Memory neural networks for spatio-temporal insights Outperforms existing models in various climates
Using natural temperature differences between air and soil [7]	Kamil Bancik, 2024	Exploration of soil-air temperature gradient for powering sensor nodes Development and testing of a TEG-based device 7852.2 J of energy harvested, demonstrating feasibility and sustainability of the solution
Energy harvesting from soil temperature differences [8]	Sven Pullwitt, 2018	Investigation of TEGs for power from soil–ground temperature differences Development/testing of a state-of-the-art TEG device Presentation of long-term energy harvesting data Demonstration of viability of soil temperature energy harvesting for outdoor use
Impact of temperature on the dynamics of organic matter [9]	Jean-Yves Cornu, 2016	Study of effects of soil temperature rise on metal bio-availability and plant absorption Soil temperature alters organic matter’s metal affinity, impacting bio-availability Important food safety concerns in warming climates

**Table 2:** Machine learning models for soil temperature prediction and their features.

Model and Description
Polynomial Regression (PR) Linear Regression chained with polynomial preprocessing [13] Alternative to Neural Nets [13,17] Fast learning, low computational demands [13]
Support Vector Regression (SVR) Regression model from the Support Vector Machine family [14] Promising performance in various scientific forecasting domains [18] Good ratio of computational requirements to model performance [14] Able to handle non-linear multidimensional problems [19]
Long-Short Term Memory (LSTM) Validated type of recurrent neural network [20] Can be widely optimised, achieves very low error rates [15] Strong performance in time series analysis [16]

**Table 3:** Various parameters used for LSTM model.

Parameter	Value
input size	depends on number of features in feature set
output size	4
output activation	sigmoid
LSTM layers	2–3 layers
LSTM units per layer	[512, 256, 128, 64]
LSTM activation	hyperbolic tangent (tanh)
LSTM recurrent activation	sigmoid
Dense layers	2–3 layers
Dense units per layer	[256, 128, 64, 32]
Dense activation	Rectified Linear Unit (ReLU)
L1 and L2 penalties	<0.0–0.05>
dropout	<0.0–0.3>
loss function	MSE
observed metrics	MAE, accuracy
optimiser	ADAM
learning rate	[0.01, 0.001]

**Table 4:** Variables measured in the input dataset.

Variable	Description	Units
*F*	wind speed	m/s
*p*	atmospheric pressure	hPa
*S*	solar irradiance	W/m^2^
*R*	precipitation	mm
*T*	ambient temperature	°C
$T_{xxx}$	soil temperature at depths of 5 cm, 10 cm, 20 cm, 50 cm, 100 cm	°C

**Table 5:** Evaluation criteria for comparing the results produced by the LSTM models.

Criteria	Description	Units
MAE	Mean Absolute Error (MAE)	°C
RMSE	Root Mean Squared Error (RMSE)	°C
$R^{2}$	R-squared ($R^{2}$), goodness of fit	-
Error Ratio	Weighted error ratio of model	%
Total Score	Calculated total score of model	%

**Table 6:** Feature sets.

Features	Reason
*T*	ambient temperature only
*T*, *S*	basic key combination between ambient temperature and solar radiation
*T*, *S*, *F*	negative weak correlation between wind and soil temperature
*T*, *S*, *p*	positive weak correlation between atm. pressure and ambient temperature
*T*, *S*, *F*, *p*	negative weak correlation between wind and atm. pressure
*T*, *S*, *R*	experimental combination
*T*, *S*, *R*, *F*	experimental combination
*T*, *S*, *R*, *F*, *p*	experimental combination of all available features

**Table 7:** Metrics for the best feature combination for the PR, SVR, and LSTM models at various depths.

Depth	Model	MAE	RMSE	$R^{2}$	TS	ER
(cm)		(°C)	(°C)	(-)	(%)	(%)
10	PR	1.19	1.48	0.959	90.61	5.55
	SVR	1.24	1.55	0.955	89.98	5.81
	LSTM	1.09	1.37	0.965	91.53	5.14
20	PR	1.20	1.49	0.957	90.13	5.83
	SVR	1.23	1.53	0.955	89.79	5.97
	LSTM	1.10	1.38	0.963	91.15	5.38
50	PR	1.12	1.36	0.957	89.68	6.31
	SVR	1.22	1.51	0.947	88.09	6.99
	LSTM	0.99	1.23	0.965	91.00	5.69
100	PR	0.92	1.13	0.962	90.26	6.14
	SVR	0.98	1.20	0.957	89.47	6.51
	LSTM	0.79	0.98	0.971	91.95	5.32

TS—Toatal score, ER—Error Ratio.

**Table 8:** Comparison of the results from the present study with the results from a reference study.

	Proposed Solution			Reference Study [5]			
Features	2			8 (12)			
Depth	MAE	RMSE	$R^{2}$	MAE	RMSE	$R^{2}$	Improvement
	(°C)	(°C)	(-)	(°C)	(°C)	$R^{2}$	(%)
10 cm	1.09	1.37	0.96	1.34	1.69	0.93	18.7
20 cm	1.10	1.38	0.96	1.24	1.55	0.93	11.3
50 cm	0.99	1.23	0.96	1.07	1.35	0.92	7.5
100 cm	0.79	0.98	0.97	0.84	1.04	0.91	6.0

## Data Availability

The data presented in this study are available on request from the corresponding author.
